# QTL-Seq and Sequence Assembly Rapidly Mapped the Gene *BrMYBL2.1* for the Purple Trait in *Brassica rapa*

**DOI:** 10.1038/s41598-020-58916-5

**Published:** 2020-02-11

**Authors:** Xin Zhang, Kang Zhang, Jian Wu, Ning Guo, Jianli Liang, Xiaowu Wang, Feng Cheng

**Affiliations:** 1 Institute of Vegetables and Flowers, Chinese Academy of Agricultural Sciences, Key Laboratory of Biology and Genetic Improvement of Horticultural Crops of the Ministry of Agriculture, Sino-Dutch Joint Laboratory of Horticultural Genomics, Beijing, China; 2 Beijing Vegetable Research Center, Beijing Academy of Agriculture and Forestry Sciences, National Engineering Research Center for Vegetables, Key Laboratory of Biology and Genetic Improvement of Horticultural Crops (North China), Ministry of Agriculture, P. R. China, Beijing, 100097 China

**Keywords:** Agricultural genetics, Genomics

## Abstract

Anthocyanins have strong antioxidant activity and are believed to be healthy for human beings. The *Brassica rapa* L. ssp. *chinensis* var. *purpurea* “Zicaitai” is rich in anthocyanins. We constructed an F_2_ population of Zicaitai and “Caixin” (*Brassica rapa* ssp. *parachinensis*) and it shows clear segregation of the purple phenotype (i.e., variation in anthocyanin enrichment). Here, quantitative trait locus (QTL)-Seq was performed with two sample groups from the F_2_ population: one exhibiting an intense purple phenotype and the other showed a completely green phenotype. The results showed that the QTL-Seq and linkage analysis located different major loci. This indicates that there are two major genetic factors that plays different roles in regulating anthocyanin enrichment in Zicaitai. This was further supported by the data simulation of an in silico F_2_ population that QTL-Seq and linkage analysis can locate different major loci. Furthermore, the draft genomes of the two parents (Zicaitai and Caixin) were assembled and utilized to search for mutations in candidate genes. A ~100-bp insertion was found in the third exon of gene *BrMYBL2.1* in Zicaitai. *BrMYBL2.1* is a negative regulator of anthocyanin biosynthesis, while *BrEGL3.2*—previously located by linkage mapping—is a positive regulator. For these populations with multiple genes contributing large effects to a trait, a strategy of low depth re-sequencing of F_2_ individuals followed by QTL-Seq analysis with the free combination of sample groups is proposed. Furthermore, draft-sequence assembly of parental genomes together with QTL mapping is suggested as an efficient means for fine-mapping genes rapidly in segregating populations.

## Introduction

Anthocyanin pigments are important flavonoid compounds that exhibit a wide range of biological functions in plants^1–3^, including as attractants for pollinators and seed dispersers and in protecting plants against abiotic and biotic stresses^4,5^. More importantly, anthocyanins show beneficial effects to human health and exhibit potential protective functions against cancer and heart disease^6,7^. These properties are partially attributed to their strong antioxidant capacity^8^. According to studies in the model plant *Arabidopsis thaliana*, regulatory anthocyanin genes can be mainly divided into positive and negative regulatory genes based on whether they promote or inhibit the expression of structural genes for anthocyanin biosynthesis, respectively^9^. Positive regulatory factors primarily include three types of genes: R2R3-MYB^10^, basic helix-loop-helix (bHLH), and WD40 transcription factors^11^. These genes promote the biosynthesis of anthocyanins. Additionally, there are two main types of negative regulatory genes, including the R3-MYB transcription factor^10,12^ and the nitrogen-induced LBD transcription factor^13^. The bioavailability and activity of anthocyanins and their regulatory genes vary widely across plants^5,14^. “Zicaitai” (*Brassica rapa* L. ssp. *chinensis* var. *utilis*), a representative purple variety of *B. rapa*, exhibits purple petioles and flower stalks^15^. The purple pigment of Zicaitai has been confirmed as anthocyanins^4^. Although several studies have characterized anthocyanins in *Brassica* crops^16–19^, there is limited information on the genes involved in anthocyanin biosynthesis in Zicaitai.

Quantitative trait locus (QTL)-Seq is a method that combines bulked segregant analysis (BSA) and high-throughput whole-genome re-sequencing to detect the major locus of a certain quantitative trait in a segregating population. BSA was proposed in 1991^20,21^. It selects parents that show a contrasting phenotype on a trait of interest to build a segregating population—either F_2_ recombinant inbred lines, double haploid, or backcross populations—and then selects two groups of individual plants, each showing segregation of the trait to one of the parents, as two mixed pools to perform genotype analysis. The genomic position of the polymorphic molecular markers that shows significant segregation of genotypes is the region that harbors the major QTL. Currently, BSA has been updated to QTL-Seq through the replacement of traditional markers such as RAPD (random amplification polymorphic DNA) or RFLP (restriction fragment length polymorphism) to SNP (single nucleotide polymorphism) markers, accompanied by high-throughput re-sequencing and SNP-index analysis^22^. QTL-Seq shows much higher efficiency than traditional QTL mapping, which is typically time-consuming and involves labor-intensive genotyping and maintenance of the mapping populations. However, QTL-Seq analysis always locates one locus for a trait at one time, while traditional QTL mapping may locate several loci for one trait in one experiment. QTL-Seq has been widely and successfully used in many crop populations, such as in the mapping of flowering traits in cucumber^23^, tomato fruit weight^14^, and 100-grain weight and root traits of chickpeas^24,25^.

In the present study, we performed QTL-Seq analysis and located different major loci to that obtained by linkage mapping using the same F_2_ population of Zicaitai and “Caixin”. A simulated population was generated and analyzed, and the results supported that QTL-Seq and linkage mapping are able to detect different major loci under the combination of different modes of inheritance. We further assembled the draft-genome sequences of Zicaitai and Caixin. By combining the assembled sequences with the locus located by QTL-Seq, we determined the causal genes and their functional mutations responsible for anthocyanin enrichment in the Zicaitai accession.

## Materials and Methods

### Plant materials and whole-genome re-sequencing

Zicaitai DH line ZCT095 was used as the receptor parent and Caixin DH line L58DH was used as the donor parent to construct an F_2_ population of 200 plants. The picture of the two parental lines have been shown in previous study^3^. 30 plants exhibiting an extreme purple phenotype were selected from the population as the purple group, while 30 non-purple plants were selected as the green group. The frequency distribution on the anthocyanin accumulation in the F_2_ population were also shown in previous report, plants that have more than 0.4 mg/g dry weight of total anthocyanin content were selected and considered as the purple group. DNA from both parents and two groups of 60 samples was extracted from fresh leaves at the six-leaf stage. The DNA samples from the two groups were then combined into two pools for library construction. Following this, 100-bp pair-end reads were generated by the Illumina Solexa sequencing platform from BerryGenomics Biotech Co., Ltd. (Beijing, China).

### Variant calling with re-sequencing data

The paired-end reads of Zicaitai, Caixin, and the two sample pools were aligned to the *B. rapa* Chiifu reference genome version 3.0 using BWA^26^ software with the method “mem”^27^. Samtools^28^ was then used to call the SNP and InDel variants from the aligned reads of Zicaitai and Caixin. Low quality (Q < 10, DP < 5), multi-allelic, or heterozygous variants in the VCF files of Zicaitai and Caixin were filtered out using Bcftools^29^. Finally, SNP datasets were searched for polymorphic loci between Zicaitai and Caixin. Following this, the genotypes of these polymorphic loci were called out from the aligned reads of the two sample pools.

### Draft-genome assembly of the two parental lines

The adaptors, duplicates, and low-quality reads were filtered from the raw Illumina Sollexa sequencing reads produced from three DNA libraries with insert sizes of 180 bp, 350 bp, and 500 bp. The filtered datasets were then submitted for assembly into contig sequences using *SOAPdenovo* (Version r240)^30^, implementing the default parameters. And then gaps in the assembly were closed using the tool GapCloser. Finally, the two parental genomes were assembled into scaffolds with N50 lengths of 4.5 kb and 6.5 kb, with a total length of 403.9 Mb and 350.8 Mb for Zicaitai and Caixin, respectively. The sequences of the draft assembly were deposited in BRAD database (http://brassicadb.org/brad/datasets/pub/Zct_Cx/assemble/).

### QTL-Seq analysis

The SNP-index was calculated for all SNPs in the two pools of mixed samples, with the genotype of Zicaitai as the reference. During the calculation, we filtered SNPs with a SNP-index <0.3 or >0.7, which denotes a co-segregation of certain genotypes in the two pools. A 200 kb sliding window with a 20 kb increment was applied to slide across the genome, and the average value of the SNP-index was calculated in each window. The Δ(SNP-index) of Zicaitai (range from −1 to +1) was calculated by using the SNP-index of the purple pool minus the SNP-index of the green pool. We further repeated these calculations with the genotype of Caixin as the reference to obtain the Δ(SNP-index) of Caixin (range from −1 to +1). Finally, the Δ(SNP-index) of the population (range from −2 to +2) was calculated from the sum of Δ(SNP-index) of Zicaitai and Δ(SNP-index) of Caixin and plotted along the 10 chromosomes of *B. rapa* to show the signals detected by the QTL-Seq analysis.

### Linkage mapping

The software MapQTL (https://www.kyazma.nl/index.php/MapQTL/) version 6 was used to perform linkage analysis with genotype datasets from both populations of Zicaitai/Caixin and the simulation. The algorithm “MQM mapping” was selected to perform the calculation. The output logarithm of odds (LOD) scores were plotted along the genetic distances of the markers analyzed.

### Population simulation and analysis

The polymorphic positions between Zicaitai and Caixin in 10 chromosomes were used as coordinates to simulate two sets of genotype data. The two genotype datasets were considered as the homologous genotypes of two homozygous parental genomes. The combination of the two sets of genotypes (two parental haplotypes) resulted in the heterozygous status of a simulated F_1_ genome. Based on this F_1_ genome, we further simulated recombination events between the two parental haplotypes. Besides the random combinations of different chromosomes between the haplotypes, we simulated ~25 recombinations with 20% bias for both the pollen and egg haplotypes. It means 2.5 recombination events for each chromosome in average. We simulated 200 such haplotypes in total for both types. One egg haplotype was then randomly combined with one pollen haplotype to generate genotypes for the 200 F_2_ individuals. Six loci (different to that of Zicaitai and Caixin population) were further set to simulate trait-related genetic factors, with one locus showing dominant suppression (epistasis) on the other five loci. While the other five loci directly contribute to the phenotype level of the simulated trait and follow the recessive genetic model, with one locus designated as the major factor contributing 60% to the phenotype, and each of the other four loci contributing 10% to the phenotype. Based on these rules and the simulated genotype data of the 200 F_2_ individuals, we obtained the phenotype levels for all F_2_ individuals. These simulated data were then submitted to both QTL-Seq and linkage analysis. The perl script for the population simulation is available through the link: http://brassicadb.org/brad/datasets/pub/Zct_Cx/simCode/.

### Determination of candidate genes

Based on the comparative genomic information between *B. rapa* and *A. thaliana*, we identified the pairwise syntenic-gene relationships between the two species. With the functional information of syntenic orthologs in *A. thaliana*, as well as our previous work on the determination of anthocyanin biosynthesis genes in *B. rapa*, we predicted the potential functions of candidate genes located in the QTL regions detected by QTL-Seq. We selected the intersection region of five sliding windows (200 kb sliding window with a 20 kb increment) that have the highest ∆SNP-index as the candidate interval, which are the peak signal in A07. Since the size of the sliding window was 200 kb and the increment was 20 kb, the intersection of five neighbor windows is 120 kb. The start and stop positions of the interval were then refined according to the locations of SNPs around its boundaries. It was found that there was one anthocyanin-synthesis related gene, *BrMYBL2.1*, located at this QTL region of A07.

### Experimental verification of a large-sequence insertion

DNA was extracted from the fresh leaves of both Zicaitai and Caixin, and a pair of primers was designed to amplify the sequence that contains the insertion variant from the two DNA samples. The PCR system constituted 20 μL of PCR mixture with 2 μL of DNA (120 ng/μL), 10 μL of 2 × Rapid Taq Master Mix from the Vazyme Biotech Co., Ltd. (Nanjing, China), 0.8 μL (10 μM) of both forward and reverse primers, and 6.4 μL of ddH_2_O. The reaction mixture was incubated in a thermal cycler (9700, ABI, USA) at 95 °C for 3 min, followed by 35 cycles of 95 °C denaturation with 15 s for each cycle, following by primer annealing at 60 °C for 15 s, and a final extension at 72 °C for 5 min. The PCR products were further separated by electrophoresis on 1% agarose gel, running at 150V for 10 min, and then submitted to silver staining for band analysis. The PCR products were then sent to Majorbio Pharmaceutical Technology Co., Ltd. (Shanghai, China) for Sanger sequencing. The sequencing results were analyzed in BioEdit (http://www.mbio.ncsu.edu/bioedit/bioedit.html) and MUSCLE^31^.

## Results

### BSA-Seq determines different major loci to those of linkage mapping

An accession of Zicaitai was crossed with an accession of Caixin to create an F_2_ population of 200 individual plants showing segregation of the trait of anthocyanin enrichment (i.e., phenotypic variation in the purple color observed on the stem and leaf). We further selected two groups of F_2_ plants exhibiting an intense purple color, with one group containing 30 individuals showing intense purple and the other containing 30 samples lacking any purple. The DNA of the two groups of samples was extracted and combined into two respective pools. The two DNA pools together with two DNA samples of the two parents Zicaitai and Caixin were sequenced on the Illumina Solexa sequencing platform. The genomes of the two parents were sequenced to ~10× coverage for each (~5-Gb Illumina re-sequencing data), while the two pools were sequenced to ~20× coverage for each (~10-Gb data; Table 1). Using the genome sequences of “Chiifu” as the reference, the reads were aligned and SNP and insertion/deletion (InDel) variants from the genomes of the two parents were called. Polymorphic loci of the SNPs and InDels between the two parents were selected for further analysis. The resequencing data of the two mixed pools were then aligned to the reference genome, and the genotypes of these polymorphic loci between the parents were called out from each of the two pools.

**Table 1:** The statistics on sequencing data generated for QTL-Seq and draft-genome assembly.

Studies	Sample	Library size (bp)	Clean data (Gb)
QTL-Seq	Zicaitai	350	5.31
	Caixin	350	5.42
	Pool 1	350	10.99
	Pool 2	350	11.01
Assemble	Zicaitai	180	21.26
	Zicaitai	500	5.46
	Caixin	180	21.40
	Caixin	500	5.62

**Figure 1:**
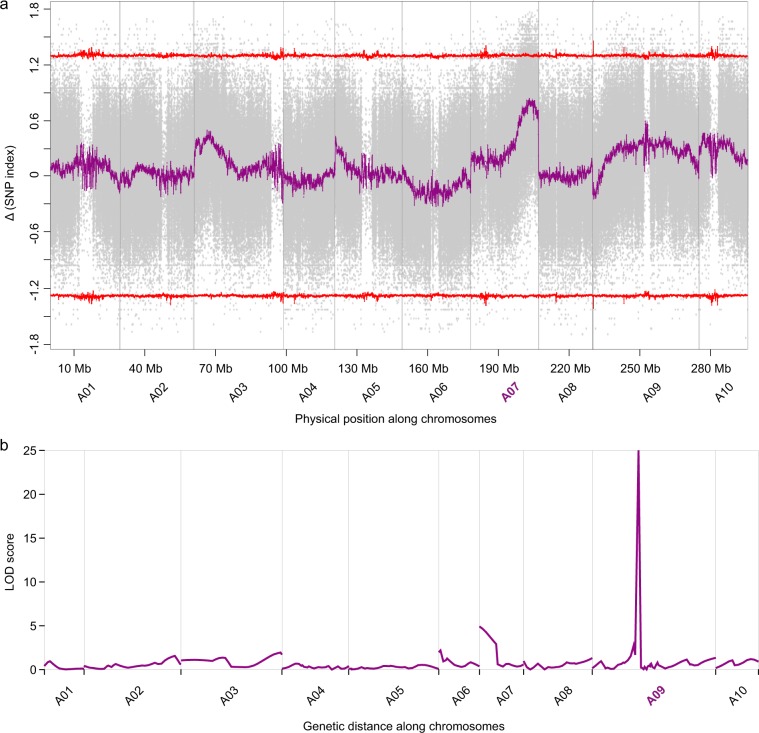
Major QTL loci of anthocyanin enrichment determined from an F_2_ population of Zicantai/Caixin. (**a**) QTL-Seq located at the major QTL locus at chromosome A07; the grey points are the Δ(SNP-index) of each SNP, the purple points are the average values of the Δ(SNP-index) in a 200-kb sliding window with a 20-kb increment across all chromosomes, and the red lines show the confidence intervals of the Δ(SNP-index). (**b**) The LOD scores generated by linkage analysis with the MEM algorithm using software MapQTL6.0. The major QTL locus was located at chromosome A09.

A significant signal was detected in chromosome A07, which differs from our previous linkage mapping results^3^. A total of 1.19 million SNPs were identified between the two parental lines. In the F_2_ pool, approximately 1.02 million SNPs were detected at the polymorphic positions between the parental lines. Using the genotype datasets of the two parents and two pools, the SNP-index and Δ(SNP-index) were calculated to locate the QTL locus (see Methods). Figure 1 shows a major locus that was detected in close proximity to the end of chromosome A07, which harbors the genetic factor that regulates the formation of extreme levels of purple color, i.e., contributes the major effect of anthocyanin enrichment. We previously performed linkage mapping with the same F_2_ population on the trait of anthocyanin variation and determined a major QTL on chromosome A09^3^, which differs from the current major location identified by QTL-Seq. Additionally, a weak QTL signal was also detected in A07, suggests that these is a genetic factor located at A07 which also contributes to the anthocyanin enrichment in Zicaitai. Together with the information of physical positions of these genetic markers used in linkage mapping, we confirmed that the two methods located distinct major loci for anthocyanin enrichment in one F_2_ population—one locates at A07, while the other one locates at A09. The results suggest the existence of different genetic factors that may adopte different models of inheritance in the regulation of anthocyanin formation in this Zicaitai accession.

### Simulation experiments for assessing the inconsistency between the QTL-Seq and linkage analysis

We generated simulation data to explore the potential possibility of the inconsistent chromosomal locations detected by QTL-Seq and linkage analysis. Firstly, two sets of genotype data were simulated on the same positions of the polymorphic SNPs between Zicaitai and Caixin and were considered as genotypes of two in silico homozygous parents. Secondly, we simulated recombination events (cross-overs) between the two parental haplotypes (i.e., half of the homozygous genotypes), as occurs in the sex cells in F_1_ plants, with one paternal haplotype (pollen) or one maternal haplotype (egg) having ~25 cross-overs (<0.2 bias). With this rule, we generated 200 such paternal and maternal haplotypes. Thirdly, we randomly selected one paternal haplotype and one maternal haplotype, and combined them into the genotype data of one simulated F_2_ plant—the process of selfing of F_1_ plants. We repeated this process and simulated 200 such F_2_ plants. Furthermore, a trait and its causal loci were simulated in one of the parents, and the information of their inheritance in the F_2_ population was surveyed and recorded for further mapping analysis. To design multiple factors regulated trait, we simulated six loci (Supplementary Table S1) that were associated to the final trait, with five loci contributing directly to the level of the trait. Among the five loci, one contributed 60% to the final phenotype of the trait and followed the recessive genetic model, while each of the other four loci contributed 10% to the phenotype of the trait and also followed the recessive model. The last locus does not contribute directly to the trait, but shows epistatic effects on all the other five loci and follows the dominant genetic model. Under these rules, one of the parents shows 100% of the trait, while the other one shows 0% F_1_ also shows 0%, which is similar to the purple phenotype in the Zicaitai-Caixin population. Combining these rules with the simulated genotypes of the F_2_ population, we further calculated the trait level in the 200 simulated F_2_ individuals.

**Figure 2:**
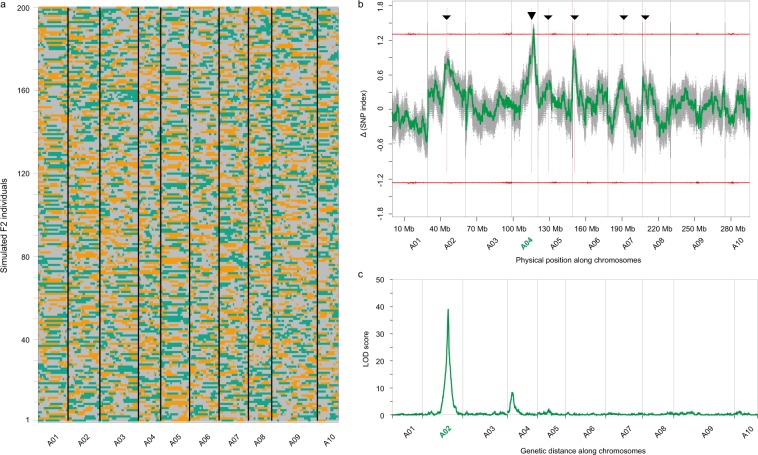
QTL mappings in a simulated population with 200 F_2_ individuals. (**a**) The inheritance of parental genomic fragments in each of the F_2_ individuals; green fragments are from parent one, orange fragments are from parent two, while grey fragments are heterozygous genotype from both parents. (**b**) QTL–Seq analysis located the major QTL locus at chromosome A04. The colors denote similar objects as that in Fig. 1. The six black triangles denote the locations of the simulated loci. The bigger triangle at chromosome A04 is epistatic to the other loci. (**c**) Linkage analysis mapped the major QTL locus at chromosome A02.

We performed linkage mapping and QTL-Seq analysis with these simulated genotype datasets and the trait information, following the same analysis pipeline as that used in studies on the real F_2_ population of Zicaitai-Caixin. Figure 2a shows the inheritance patterns of the parental genomic fragments in each of the 200 F_2_ individuals. Linkage analysis identified a major locus on chromosome A02 (Fig. 2c), which corresponds to the simulated epistatic locus. Another smaller signal was detected on A04, which corresponds to the simulated major locus (60% contribution) that directly regulates the simulated trait. Additionally, we selected two groups from the F_2_ population, with one group of samples showing >70% of the trait and the other showing 0% of the trait. Each group contained 25 samples and were recorded as a pool. We further simulated ~30 reads to cover the simulated SNP loci of the 25 samples in each pool and performed QTL-Seq analysis using the same method as that used in the real Zicaitai-Caixin population. The result showed that the QTL-Seq located a major locus at chromosome A04 (Fig. 2b), corresponding to the simulated major locus (60% contribution)—which is different to the major locus (chromosome A02) mapped by linkage analysis. This simulation analysis generated similar inconsistence between QTL-seq and linakge mapping analysis as that observed in the mapping of anthocyanin enrichment trait in the Zicaitai-Caixin population, supporting that there are two different loci following different genetic models relating to purple color regulation in Zicaitai.

### Draft-sequence assembly and fine-mapping of the causal gene

**Figure 3:**
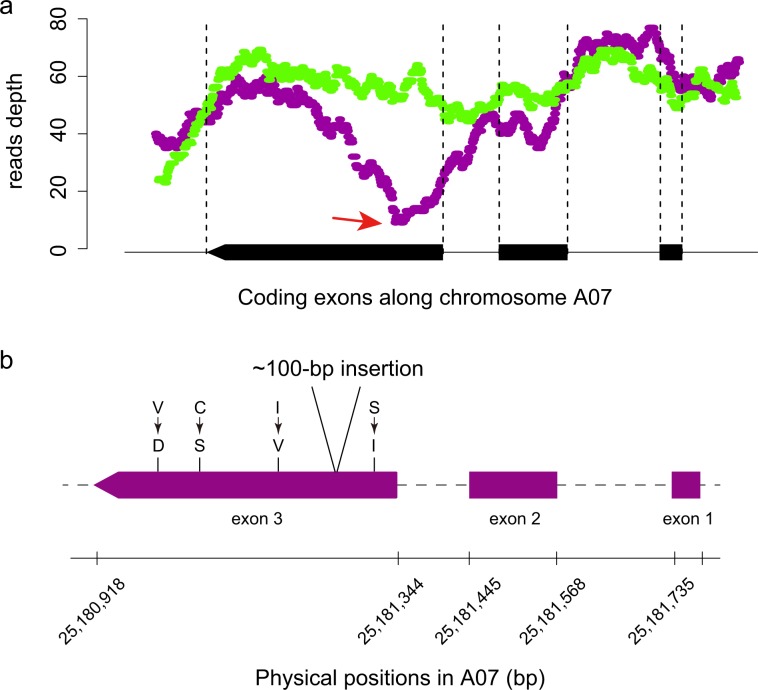
A large sequence insertion was found in gene *BrMYBL2* in Zicaitai. (**a**) The depth the reads covered on each nucleotide around the genomic region of gene *BrMYBL2*; purple points denote the reads depth from the sequencing data of purple pool in the QTL-Seq analysis, while green points denote reads depth from that of the green pool. (**b**) The gene model of *BrMYBL2* and the positions of a sequence insertion and four non-synonymous mutations in the third coding exon of this gene in Zicaitai.

We screened candidate genes in the mapped regions with the annotation information and functional mutations of both genes. We previously predicted the anthocyanin biosynthesis genes in the genome of *B. rapa* by comparison with genes in *A. thaliana*^32^. As reported in the linkage analysis, there are two such genes, *BrEGL3.1* (*BraA09g015240.3C*) and *BrEGL3.2* (*BraA09g013410.3C*), in the QTL region of A09^3^ (Table 2). They are two homeologs of the EGL gene, which is a positive regulator of anthocyanin biosynthesis in *A. thaliana*. Furthermore, we found one gene, *BrMYBL2.1* (*BraA07g036130.3C*), located almost at the midpoint of the major locus in A07 (Table 2), which was detected by QTL-Seq. *BrMYBL2.1* is a homeolog of the MYBL2 gene, which, in contrast to EGL3, is a negative regulator of anthocyanin biosynthesis. We further assessed the sequencing depth of the *BrMYBL2.1* gene to determine if there was any structural variant that could not be determined by variant calling based on the re-sequencing reads mapping. By comparing the differences in sequencing depth between the two pools of purple and non-purple plants, we detected that in the third exon of *BrMYBL2.1*, a position in the purple pool exists that shows much lower sequencing depth than the non-purple pool (Fig. 3a).

**Table 2:** Candidate anthocyanin-biosynthesis genes in major QTL loci.

QTL loci			Candidate genes		
Chromosome	Start	Stop	ID	Start	Stop
A07	25,112,903	25,230,669	*BrMYBL2.1*	25,180,918	25,181,774
A09	7,230,396	9,242,789	*BrEGL3.1*	8,904,764	8,908,450
			*BrEGL3.2*	7,821,182	7,824,280

We assembled draft genome sequences of Zicaitai and Caixin to investigate the structural mutations in the candidate genes of anthocyanin enrichment between the parents. For both Zicaitai and Caixin, we generated ~80× coverage sequencing data in total (Table 1). These data was used to assemble the two genomes. Finally, we obtained draft genome sequences for both Zicaitai and Caixin with assembled sizes of ~403-Mb and ~350-Mb, and scaffold N50 values of 4-kb and 6-kb, respectively (Table 3). Although the two assembled genomes do not possess sufficient sequence contiguity, they are useful for the detection of structural variations in the coding sequence regions of the genes. Assembled sequences were then compared with candidate genes to assess for structural variations. We BLASTed the scaffolds of both Zicaitai and Caixin against the coding sequences of the candidate genes. The structural variations between Zicaitai and Caixin on each of these candidate genes were then observed on these aligned sequences. By doing this, we determined a large sequence insertion (with “N” as a gap in it) in the third exon of *BrMYBL2.1* in Zicaitai compared to that of Caixin, which overlapped with the region that shows lower re-sequencing depth in the purple pool than the non-purple pool (Fig. 3b). No such structural variations were found in the other candidate genes.

**Table 3:** The draft-genome-assembly information of the two parents.

Sample	Assembled size (Mb)	Max length (bp)	N50 (bp)
Zicaitai	403.95	98,182	4,576
Caixin	350.81	95,917	6,543

### Mutation verification through Sanger sequencing

**Figure 4:**
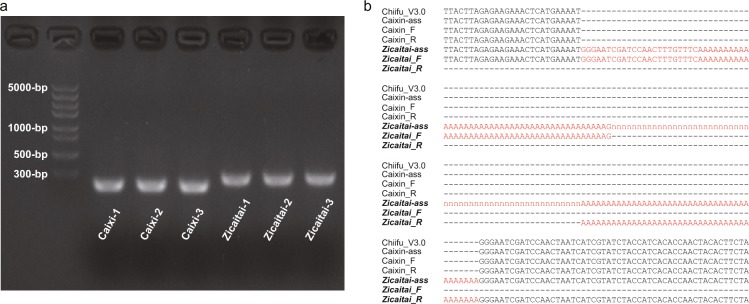
Experimental verification of a sequence insertion in gene *BrMYBL2*. (**a**) The electrophoretogram of the PCR products of the target sequence that contains the insertion variant. Three replicates for both Caixin and Zicaitai were labeled as one to three. (**b**) The alignment of the target sequences from the Chiifu reference genome (Chiifu_V3.0), draft sequence assembly of Caixin (Caixin-ass) and Zicaitai (Zicaitai-ass), and Sanger sequencing results (Caixin-F and Caixin-R, Zicaitai-F and Zicaitai-R, F denotes forward sequence, R denotes reverse sequence). The red color denotes the inserted sequence.

To verify the insertion variation in *BrMYBL2.1* of Zicaitai, we performed polymerase chain reaction (PCR) amplification of the sequence containing the insertion. The electrophoresis products indicated that the size of the amplified sequences differed between the two parents. The sequence amplified from Zicaitai was ~100-bp longer (estimated based on the size of the DNA ladder) than that of Caixin (Fig. 4a), which is consistent with previous results of the comparisons between the assembled sequences. The PCR products were further submitted to Sanger sequencing, and the results showed that there was a sequence insertion containing “GGGAATCGATCCAACTTTGTTTC” linked with poly-“A”s to the gene in Zicaitai (Fig. 4b) in comparison with Caixin. This confirmed the sequence insertion detected by the sequence assembly. Sanger sequencing peaks denoting different types of nucleotides were observed to be overlapping with each other following the poly-A and could not be easily recognized (high level of heterozygous peaks) for determining the accurate size and sequences of the insertion variation. However, the ~100-bp sequence insertion in the third coding exon of *BrMYBL2.1* changed the coding protein in Zicaitai. The gene *BrMYBL2.1* in chromosome A07 is a negative regulator of anthocyanin biosynthesis, while *BrEGL3.1*, located at the QTL region of A09, is a paralog of EGL3, which is a positive regulator of anthocyanin biosynthesis. The functional mutations of the two genes both contributed to the variation in anthocyanin enrichment of Zicaitai. The different roles of the two genes in the biosynthesis of anthocyanins explains the different results of the QTL-Seq and linkage mapping, even when using the same population.

## Discussion

QTL-Seq and linkage analysis using the same segregating population can identify different major loci. In this work, we performed QTL-Seq analysis on the purple phenotype (i.e., the trait of anthocyanin enrichment) in an F_2_ population of Zicaitai-Caixin and compared the result with our previous linkage-analysis study using the same population. The two methods located different major anthocyanin enrichment loci in Zicaitai, which indicated that two major genetic factors contribute to the trait and have different roles and/or follow different genetic models in the regulation of anthocyanin enrichment. In order to test this, we generated a simulated F_2_ population with two major loci regulating a trait under different genetic models. The first directly contributes to the phenotype under a recessive model, while the second is epistatic to the first locus and follows a dominant model. QTL-Seq and linkage mapping were then conducted on the simulated population using the same pipeline as that applied to that of the Zicaitai-Caixin population. The results showed that QTL-Seq and linkage mapping located different major loci from that observed in the Zicaitai/Caixin population. QTL-Seq located the major locus that regulates the trait directly, while the linkage mapping located the locus that is epistatic to the previous locus. It is reasonable that the sampling of two pools with opposite and extreme traits will cause the QTL-Seq to detect a locus associated with a high level of the trait, while linkage analysis will locate the gene that shows a clearer inheritance pattern. Inspired by the simulation analysis, we speculated that these two major factors identified through QTL-Seq and linkage mapping in Zicaitai-Caixin F_2_ populations should have different regulation pathways and/or follow different genetic models relating to anthocyanin biosynthesis. Actually, the *BrMYBL2* gene detected by QTL-Seq function as a negative regulator and may contribute directly to the purple trait in Zicaitai. While in the linkage analysis, the positive regulator gene *EGL3* with potentially epistatic function was localized to mediate the anthocyanin biosynthesis.

The complexity of the regulation mechanisms on anthocyanin biosynthesis in *Brassica*s were highlighted by recent studies. Many efforts have been devoted to dissect the genes that contribute to the purple traits in different *Brassica* crops. In *Brassica napus*, *BnAPR2*, encoding an adenosine 5’-phosphosulfate reductase, at the end of A03 chromosome was identified as an incomplete dominant regulatory gene through map-based cloning^33^. Another gene *BrMYB73* was also mapped to the end of A03 in *B. rapa*^34^. *BrMYB73* was predicted to encode a R2R3-MYB transcription factor, and one deletion and one SNP were found in this gene in purple-leaf parent. Recently, Li *et al*. identified a *bHLH49* transcription factor (*BrbHLH49*) on A07 that might positively regulate the anthocyanin accumulation in Zicaitai based on a specific-locus amplified fragment sequencing method^19^. These studies revealed the genetic diversity of anthocyanin biosynthesis related genes in *Brassica* crops.

QTL mapping together with draft genome assembly is an efficient means of fine-mapping causal genes and mutations from segregating populations. In QTL analysis, considering the limited recombination events in a given population, the mapping resolution is not always sufficient to locate the causal genes, and dozens or hundreds of candidate genes can make the fine-mapping of the gene difficult. Furthermore, with variants called from the re-sequencing data, only small-scale variants, such as SNPs and short InDels, can be analyzed in these candidate genes, which results in the major loss of large-scale functional mutations during the screening of candidate genes. However, the draft genome assembly of parental genomes and pairwise comparisons of coding sequences of candidate genes between parents will capture these large-scale mutations, thus resulting in the rapid fine-mapping of the causal genes and mutations of the traits. In this work, we performed draft genome assembly of Zicaitai and Caixin. Together with the mapping results from the QTL-Seq, we located a large sequence insertion in the coding exon of an anthocyanin biosynthesis-related gene. The insertion changed the sequence of the translated protein of the genes in Zicaitai in comparison to Caixin. Considering that high-throughput sequencing is becoming increasingly affordable, QTL mapping accompanied by the draft sequence assembly of parental genomes could constitute an efficient means of fine-mapping the genes in segregation populations.

Low-depth re-sequencing of individual samples from a segregating population is a superior option for analyzing a trait that is regulated by more than one major locus. In this study, we located two genomic regions that contribute to the enrichment of anthocyanins in Zicaitai; one being a positive regulator of anthocyanin biosynthesis and the other being a negative regulator of anthocyanin biosynthesis. The two genes have different roles in anthocyanin enrichment in Zicaitai. In similar cases, QTL-Seq as well as linkage mapping may not be able to locate all of these major loci in one experiment. Therefore, we propose a strategy of low-depth re-sequencing of all F_2_ individuals. With the re-sequencing data, a genetic map with a high density of bin markers (combined markers in a local region) can be constructed, allowing for efficient and simple linkage mapping. More importantly, different samples can be repeatedly combined into two extreme pools based on different rules, and QTL-Seq can be performed as many times as needed. This strategy should help to locate multiple major loci that contribute to one or more traits in a segregating population.

## Supplementary information

Supplementary Table S1.
