# Supplementary material for: QTL-Seq and Sequence Assembly Rapidly Mapped the Gene BrMYBL2.1 for the Purple Trait in Brassica rapa
Source: Sci Rep. 2020 Feb 11;10:2328. doi: 10.1038/s41598-020-58916-5 (PMC7012920; doi:10.1038/s41598-020-58916-5)
Supplement: Supplementary file 1 — Supplementary Table S1. [file 41598_2020_58916_MOESM1_ESM.docx]

**QTL-Seq and Sequence Assembly Rapidly Mapped the Gene *BrMYBL2.1* for the Purple Trait in *Brassica rapa***

Xin Zhang^1,＃^, Kang Zhang^1,＃^, Jian Wu^1^, Ning Guo^2^, Jianli Liang^1^, Xiaowu Wang^1,^ *, and Feng Cheng^1,^ *

^1^ Institute of Vegetables and Flowers, Chinese Academy of Agricultural Sciences, Key Laboratory of Biology and Genetic Improvement of Horticultural Crops of the Ministry of Agriculture, Sino-Dutch Joint Laboratory of Horticultural Genomics, Beijing, China.

^2^ Beijing Vegetable Research Center, Beijing Academy of Agriculture and Forestry Sciences, National Engineering Research Center for Vegetables, Key Laboratory of Biology and Genetic Improvement of Horticultural Crops (North China), Ministry of Agriculture, P. R. China, Beijing 100097, China

^＃^These authors contributed equally to this work

^*^ Correspondence should be addressed to: chengfeng@caas.cn, wangxiaowu@caas.cn

**Supplementary Table S1**. Genomic locations of the simulated loci in the simulation analysis

| #index | Chromosome | Position | Contribution(%) |
| --- | --- | --- | --- |
| 1 | A02 | 17114836 | epistatic |
| 2 | A04 | 18439946 | 60 |
| 3 | A05 | 10851161 | 10 |
| 4 | A06 | 1436770 | 10 |
| 5 | A07 | 10312985 | 10 |
| 6 | A08 | 1030166 | 10 |
